# Cross Sectional Survey of Canine Idiopathic Epilepsy Management in Primary Care in the United Kingdom

**DOI:** 10.3389/fvets.2022.907313

**Published:** 2022-06-20

**Authors:** Sebastian Griffin, Fabio Stabile, Luisa De Risio

**Affiliations:** ^1^Vet4Life Teddington, Part of Linnaeus Veterinary Limited, Teddington, United Kingdom; ^2^Southfields Veterinary Specialists, Part of Linnaeus Veterinary Limited, Basildon, United Kingdom; ^3^Linnaeus Veterinary Limited, Solihull, United Kingdom; ^4^Nottingham Trent University, Nottingham, United Kingdom

**Keywords:** dog, first opinion, veterinary, antiepileptic drugs, epileptic seizures

## Abstract

The aims of this study are to gain insight on how primary care veterinarians in the UK diagnose and treat canine idiopathic epilepsy (IE) and what they perceive as challenges in the management of canine IE. Two hundred and thirty-five primary care veterinarians took part in this survey. The questionnaire asked about the type of practice the respondent worked in, any relevant post-graduate qualifications, how many years' experience they had in practice and the participant's canine IE caseload. Participants were asked how they diagnose canine IE, how they select antiseizure drugs (ASDs) and how they assess outcome. The questionnaire also explored which information sources they have access to for deciding on canine IE treatment, challenges that may be faced when managing these cases and areas in which more support can be provided. 94.5% of participants (*n* = 222/235) managed <10 canine IE cases in a year and 87.8% (*n* = 206/235) used phenobarbital as their first line ASD. The reported mean initial phenobarbital dose was 2.1 mg/kg (standard deviation = 0.71) every 12 h. When considering how closely participants aligned with IVETF guidelines on the topics of diagnosis, ASD initiation and outcome assessment, on average participants would score around half of the available points. 53.2% (*n* = 125/235) of respondents recommended neutering in canine IE and 46.8% (*n* = 110/235) did not. 53.2% (*n* = 125/235) did not recommend any additional treatments for canine IE beyond use of ASDs. 23.4% recommended Purina Neurocare diet (*n* = 55/235), 12.8% recommended environmental modification (*n* = 30/235), and 6.8% (*n* = 16/235) recommend medium chain triglyceride supplements. In this study participants found managing client expectations to be most challenging alongside canine IE emergency management. The main limitation of this study is the relatively low response rate and therefore the results may not reflect the entire small animal veterinary profession in the UK. However, the results of this study represent a starting point to inform educational resources and support strategies to improve quality care of canine IE in primary care.

## Introduction

Canine idiopathic epilepsy (IE) is the most common chronic neurologic disease in dogs, with an estimated prevalence of 0.6–0.75% in the general canine population (1, 2). Breed specific prevalence is often much higher, being as high as 20% in Belgian shepherds in the US and 33% in certain breeding lines in Denmark (3).

Cases of canine IE are diagnosed at a young age and the disorder is often lifelong, with remission rarely achieved (4, 5). Canine IE is a complex brain disease with a broad range of impacts upon quality of life of affected dogs as well as their caregivers (6–10). Dogs with IE not only suffer from recurrent epileptic seizures but can also develop behavioral and cognitive co-morbidities (6–9). In addition, canine IE had been shown to threaten the lifespan of affected dogs (11).

Consensus statements on the diagnosis, treatment and treatment outcome of canine IE based on available scientific evidence were published in 2015, by the International Veterinary Epilepsy Task Force (IVETF) (12–14). The diagnosis of canine IE can be achieved according to 3 consequential tiers of confidence level, from lower (Tier I) to higher (Tier III), depending on available historical information, such as the age at epileptic seizure onset, normal interictal general and neurological examinations, and results of diagnostic investigations to exclude toxic, metabolic, and structural brain disease. The knowledge of genetically related dogs also affected by IE would support the confidence of the diagnosis further (12). The administration of anti-seizure drugs (ASDs) is currently the pillar of the treatment of canine IE supported by administration of a balanced consistent diet, environmental modifications, and trigger avoidance (should trigger be identified) (13). Systematic reviews and metanalyses of tolerability and efficacy of ASDs for canine IE have been published in 2014 and 2016 (15, 16). Treatment success has been defined as seizure freedom for a time span exceeding three times the longest pre-treatment inter-seizure interval and for a minimum of 3 months. Partial therapeutic success is defined as one or more of the following: prevention of cluster seizures or status epilepticus; a relevant reduction of seizure frequency based on the pretreatment seizure frequency; a reduction in seizure severity (14).

Historically the first line ASDs for canine IE included phenobarbital (authorized for use in dogs in the UK in 2001^1^ and/or potassium bromide (authorized for use in dogs in the UK in 2010^2^ due to their widespread availability and low cost. In 2013 imepitoin was introduced to the UK market^3^for management of recurrent single seizures in canine IE (17). Whilst yet not licensed for the treatment of canine IE, other ASDs with varying efficacy such as levetiracetam, zonisamide, felbamate, topiramate, gabapentin and pregabalin appear to be safe for use in canine IE and may be used as a second- or third-line ASDs under the Cascade in the UK (15, 18, 19).

It is unknown if and how much primary care veterinarians are aware of the currently published guidelines on diagnosis and treatment of canine IE and if this knowledge translates into clinical practice. Currently no study had investigated the prescribing preferences of ASDs by primary care veterinarians in the UK. Information about the use of ASDs has been investigated in other country-specific studies in Australia (18), Florida (19) and the Netherlands (20), providing useful insight in the use of ASDs for canine IE. The prescribing regulations in these countries differ from those in the UK.

The aims of this study are to gain insight on how primary care veterinarians in the UK diagnose and treat canine IE. This study investigates the challenges encountered by the primary care veterinarians in the treatment of canine IE to determine the need for support/educational material to improve patient and customer care as well as veterinarian confidence in the management of this condition.

## Materials and Methods

This is a prospective cross-sectional survey-based study into the management of canine IE and prescribing of ASDs in the UK by primary care veterinarians. The study (including the participants' information sheet and questionnaire) was approved by the Ethics Review Panel of the Royal College of Veterinary Surgeons (2021-75-GRIFFIN, approval granted 1st of October 2021).

The study used an online 18 question questionnaire plus one question to gain informed consent and one question to ascertain eligibility (Table 1). Responses were anonymous and only one response per participant was allowed. The questionnaire was developed by two experienced neurologists (one of which is a founding member of the IVETF), a primary care veterinarian and an experienced biostatistician. The questionnaire was piloted on four primary care veterinarians (not included as respondents or authors in this study) with various level of experience and expertise, including one with expertise in education and one with expertise in psychology.

**Table 1:** Questionnaire used in the cross-sectional survey of canine idiopathic epilepsy management in primary care in the United Kingdom.

**Question number**	**Question**	**Answer options**
1	I understand participation in this study is voluntary and anonymous, by completing and submitting this questionnaire I consent to participate in this study.	Tick box for consent
2	I confirm that I am a Member of the Royal College of Veterinary Surgeons (MRCVS) working in primary care in the United Kingdom (UK) and I have managed at least one dog with idiopathic epilepsy in the past 12 months.	Tick box to confirm eligibility
3	Do you currently work in corporate owned or independent owned practice?	a) Corporate b) Independent c) Prefer not to say
4	Which type of primary care practice do you work in most of the time?	a) Small animal first opinion—with 24 h care b) Small animal first opinion—without 24 h care c) Mixed practice—with 24 h care d) Mixed practice—without 24 h care e) Other (open text field to complete)
5	Do you hold any postgraduate qualification in neurology or neuroscience? (Select all that apply).	a) No, I do not hold any postgraduate qualification in neurology or neuroscience b) PGCert c) CertAVP d) Master of philosophy e) Master of research f) Master of science g) PhD h) Other, please specify
6	How many years have you been working as a vet in clinical practice?	(open text field to complete)
7	Based on which of the following criteria do you diagnose canine idiopathic epilepsy most commonly (Select all that apply)?	a) Age at the time of the first observed epileptic seizure in the following range a. 6 months−6 years b. 1–5 years c. 1–10 years b) History of one or more epileptic seizures c) No significant abnormalities on haematology and serum biochemistry d) No significant abnormalities on urinalysis e) Unremarkable physical and neurologic examination between epileptic seizures f) Unremarkable magnetic resonance imaging of the brain g) Unremarkable cerebrospinal fluid analysis h) Other (open text field to complete)
8	Approximately how many new cases of canine idiopathic epilepsy have you treated in the last 12 months?	a) <10 b) 11–25 c) 26–50 d) > 50
9	When do you initiate antiepileptic treatment in canine idiopathic epilepsy (most commonly)? (Select all that apply).	a) After 1 epileptic seizure lasting <5 min b) After 1 epileptic seizure lasting > 5 min (status epilepticus) c) After 2 or more epileptic seizures in 24 h (cluster seizures) d) After 2 or more epileptic seizures in the last month e) After 2 or more epileptic seizures in the last 3 months f) After 2 or more epileptic seizures within the last 6 months g) Other (open text field to complete)
10	Which ONE of the following anti-epileptic medications do you most commonly prescribe as a first line long term maintenance treatment in canine idiopathic epilepsy?	a) Imepitoin (Pexion) b) Levetiracetam (Desitrend, Keppra) c) Phenobarbital (Epiphen, Epityl, Phenoleptil, Soliphen) d) Potassium Bromide (Bromilep, Epilease, Libromide) e) Zonisamide (Zonegran) f) Other (open text field to complete)
11	Which initial dosage (dose in mg/kg and administration frequency in hours) do you commonly prescribe of the long-term maintenance anti-epileptic medication selected in the above question?	(open text field to complete)
12	Do you use intermittent pulsed treatment with Levetiracetam (e.g., 60 mg/kg once followed by 20 mg/kg every 8 h for 2–3 days or similar protocol) alongside one of the long-term maintenance anti-epileptic medications selected in question 10?	a) No, I do not use pulsed treatment with Levetiracetam b) Yes, I often use pulsed treatment with Levetiracetam in dogs with idiopathic epilepsy that tend to have cluster seizures c) Yes, I occasionally use pulsed treatment with Levetiracetam in dogs with idiopathic epilepsy that tend to have cluster seizures d) Other (open text field to complete)
13	Where do you most commonly find information about canine idiopathic epilepsy treatment? (Select all that apply).	a) Advice from other primary care veterinarians b) Advice from a neurology specialist c) Advice from technical advisors of the relevant pharmaceutical company d) Internet search engine (specify which one) e) Neurology book/s f) Non-peer-reviewed veterinary literature (e.g., Veterinary Times, etc) g) Peer-reviewed veterinary literature (e.g., BMC Vet Research, JASAP, Vet Rec, JVIM, etc) h) Practice policy i) Social media j) University education k) Other (open text field to complete)
14	Based on which criteria do you establish if the antiepileptic treatment is successful? (Select all that apply).	a) ≥ 50% decrease in epileptic seizure frequency and/ or decreased severity of epileptic seizures after 6 months of treatment b) ≥ 50% decrease in seizure frequency and/ or decreased severity of epileptic seizure after 3 months of treatment c) ≥ 50% decrease in seizure frequency and/ or a decreased severity of epileptic seizure, following treatment for 2–3 times the longest pre-treatment epileptic seizure free interval (e.g., for a dog with a pre-treatment longest seizure-free interval of 6 weeks, the initial post-treatment follow-up duration will be 18 weeks) d) Antiepileptic medication serum concentration within the reference range e) Client satisfaction f) Good quality of life for the dog based on assessment of its owner/ caregiver g) Lack of or minimal adverse effects h) Epileptic seizure freedom for 3 months or 3 times the longest pre-treatment epileptic seizure free interval (whichever is the longer period) i) Other (open text field to complete)
15	Which ONE of the following anti-epileptic medications do you most commonly prescribe as a second line long term treatment in canine idiopathic epilepsy?	a) Imepitoin (Pexion) b) Levetiracetam (Desitrend, Keppra) c) Phenobarbital (Epiphen, Epityl, Phenoleptil, Soliphen) d) Potassium Bromide (Bromilep, Epilease, Libromide) e) Zonisamide (Zonegran) f) Other (open text field to complete)
16	Do you recommend any other treatments for canine idiopathic epilepsy? (Select all that apply).	a) None b) Acupuncture c) Cannabinoids d) Herbal Medicine (Please specify which product below) e) Environmental modification f) Homeopathy (Please specify which product below) g) Medium chain fatty acid (MCT) supplement (Please specify which product below) h) Nutraceuticals (Please specify which product below) i) Purina Neurocare diet j) Other diet, please specify which diet k) Physical therapy l) Omega 3 Fatty Acids m) Skullcap and valerian n) Other (open text field to complete)
17	Do you recommend neutering dogs with idiopathic epilepsy?	a) Yes b) No
18	Which aspects of management of canine idiopathic epilepsy do you find the most challenging? (Select all that apply).	a) Diagnosis b) Emergency treatment of cluster epileptic seizures and status epilepticus c) Long term routine antiepileptic treatment administration, monitoring and modulation d) Knowing when to refer e) Management of client expectations f) Idiopathic epilepsy-associated behavioral changes and/or cognitive dysfunction (which can occur in some dogs during the interictal period) g) Other (open text field to complete)
19	In which area/s of canine idiopathic epilepsy would you like more educational support? (Select all that apply).	a) Diagnosis b) Emergency treatment of cluster epileptic seizures and status epilepticus c) Long term routine antiepileptic treatment administration, monitoring and modulation d) Managing client expectations e) Management of idiopathic epilepsy-associated behavioral changes and/or cognitive dysfunction (which can occur in some dogs during the interictal period f) Educational resources for owners/ carers of dogs with idiopathic epilepsy g) Emotional support for owners/ carers of dogs with idiopathic epilepsy h) Other (open text field to complete)
20	On a scale from 1 (not needed) to 7 (extremely beneficial) how beneficial would it be for you to be able to discuss canine epilepsy cases with a specialist in veterinary neurology?	Open text field to complete with selected score (1–7)

The inclusion criteria for participation in this study were: being a member of the Royal College of Veterinary Surgeons (i.e., being licensed to practice veterinary medicine in the UK), working in primary care veterinary practice in the UK, and having treated at least one case of canine IE in the previous 12 months before completing the questionnaire.

Participants were recruited via internal advertisement within Linnaeus Veterinary Ltd, other veterinary groups in the UK, and large charities providing veterinary care to dogs in the UK, as well as via social media advertisement on Twitter, Facebook, and LinkedIn. Articles inviting UK registered veterinarians to complete the questionnaire were published in various UK veterinary journals (online and/ or in print) including, Veterinary records, VetTimes, Vet Report, Vet Click, vetsurgeon.org, Improve International and Vetpol. The study was advertised repeatedly between 6th October 2021 and 30th January 2022.

The questionnaire explored the type of practice the participant worked in (i.e., corporate vs. independent, small animal vs. mixed practice, 24-h care or not), any relevant post-graduate qualifications, how many years' experience they had in practice and the participant's canine IE caseload in the 12 months prior to completing the questionnaire. This was categorized as <10, 11–25, 26–50, 50+ cases. This demographic information was used to create categories to compare against responses to the other questions.

The participants could select multiple answers to questions which related to aspects of IE diagnosis, ASD initiation and assessment of treatment success. To enable analysis of responses to questions 7, 9,14, the answers to each option were scored (from −1 to +1) based on how accurately the selected answer aligned with IVETF consensus statements (12–14). A score of +1 indicated the selection of an answer as indicated in IVETF consensus statements, a score of +0.5 indicated the selection of an answer partially aligned with what indicated in IVETF consensus statements, a score of 0 indicated an answer neither aligned or opposite to the IVETF consensus. A score of −1 indicated the selection of an answer in contradiction with IVETF consensus statements. A combined score was then calculated for each respondent reflecting how closely their answers reflected IVETF guidelines.

The scores were assigned as follows (indicated by the number in parenthesis after each possible answer):

Question 7:

Based on which of the following criteria do you diagnose canine idiopathic epilepsy most commonly (Select all that apply)?

1. Age at the time of the first observed epileptic seizure in the following range 6 months−6 years (1).
2. 1–5 years (0.5).
3. 1–10 years (-1).
4. History of one or more epileptic seizures (1).
5. No significant abnormalities on haematology and serum biochemistry (1).
6. No significant abnormalities on urinalysis (1).
7. Unremarkable physical and neurologic examination between epileptic seizures (1).
8. Unremarkable magnetic resonance imaging of the brain (1).
9. Unremarkable cerebrospinal fluid analysis (1).

Question 9:

When do you initiate antiepileptic treatment in canine idiopathic epilepsy (most commonly)? (Select all that apply)

1. After 1 epileptic seizure lasting <5 minutes (0).
2. After 1 epileptic seizure lasting > 5 minutes (status epilepticus) (1).
3. After 2 or more epileptic seizures in 24 hours (cluster seizures) (1).
4. After 2 or more epileptic seizures in the last month (0.5).
5. After 2 or more epileptic seizures in the last 3 months (0.5).
6. After 2 or more epileptic seizures within the last 6 months (1).

Question 14:

Based on which criteria do you establish if the antiepileptic treatment is successful? (Select all that apply)

1. ≥50% decrease in epileptic seizure frequency and/ or decreased severity of epileptic seizures after 6 months of treatment (0).
2. ≥50% decrease in seizure frequency and/ or decreased severity of epileptic seizure after 3 months oftreatment (1).
3. ≥50% decrease in seizure frequency and/ or a decreased severity of epileptic seizure, following treatment for 3 times the longest pre-treatment epileptic seizure free interval (e.g., for a dog with a pre-treatment longest seizure-free interval of 6 weeks, the initial post-treatment follow-up duration will be 18 weeks) (1).
4. Antiepileptic medication serum concentration within the reference range (1).
5. Client satisfaction (0.5).
6. Good quality of life for the dog based on assessment of its owner/caregiver (0.5).
7. Lack of or minimal adverse effects (1).
8. Epileptic seizure freedom for 3 months or 3 times the longest pre-treatment epileptic seizure free interval (whichever is the longer period) (1).

The scoring technique based on IVETF guidelines was used to score the questions about which criteria participants used to diagnose canine IE (maximum score 7), criteria used to decide to initiate ASDs (maximum score 4), and criteria to establish if treatment with ASDs has been successful (maximum score 6). The questionnaire also explored which information sources they have access to for deciding on canine IE treatment, challenges that may be faced when managing these cases and areas in which more support can be provided.

Participants were asked on a scale from 1 (not needed) to 7 (extremely beneficial) how beneficial would it be for them to be able to discuss canine IE cases with a specialist in veterinary neurology.

Whenever applicable, multiple-choice questions included a free text field available as “Other” to complete if a participant felt more information was required or the available options did not reflect their choices.

Because of the nature of the responses, non-parametric statistical methods were used throughout. Kruskal Wallis tests were used to assess statistical significance between categorical variables where the response was continuous or ordinal. Spearman Rank Correlation was used to assess statistical significance where both variables were continuous or ordinal. Fisher's Exact Test was used to assess statistical significance where both variables were categorical.

## Results

### Demographic Details

Two hundred and 35 veterinarians who worked in primary care in the UK took part in this survey. 72.8% (*n* = 171/235) worked in corporate practice, 26.4% (*n* = 62/235) worked in independent practice and 0.8% (*n* = 2/235) preferred not to say. 36.6% (*n* = 86/235) worked in small animal first opinion practice with 24-h care, 54.9% (*n* = 129/235) worked in small animal first opinion practice without 24-h care and 8.5% (*n* = 20/235) worked in mixed practice (with or without 24-h care).

98.3% of participants (*n* = 231/235) did not hold any post-graduate qualification in neurology or neuroscience. Therefore, this variable was not assessed statistically.

**Figure 1:**
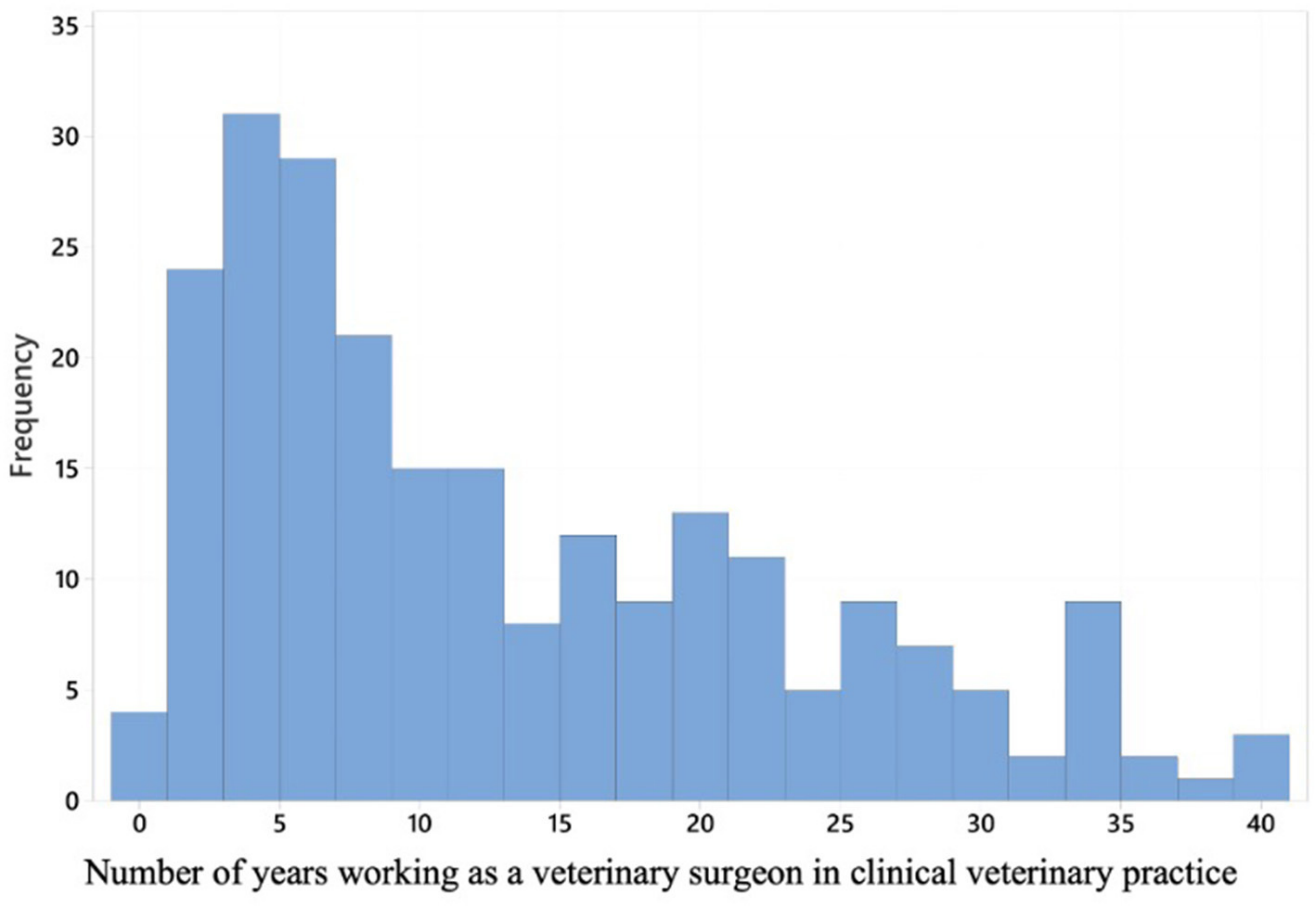
A histogram displaying years' experience working in clinical veterinary practice for the 235 respondents.

The mean number of years working as a veterinarian in clinical practice was 12.8 (standard deviation of 10.0) (Figure 1). When comparing small animal first opinion practice with 24-h care, small animal first opinion practice without 24-h care and mixed practice there was no statistically significant difference in numbers of years working as a veterinarian in clinical practice (*p* = 0.67).

94.5% of participants (*n* = 222/235) treated <10 new cases of canine IE and 5.5% (*n* = 13/235) had treated 11–25 new cases in the 12 months prior to undertaking the study survey. Of veterinarians working in independent practice 11.3% (*n* = 7/62) treated 11–25 new cases of canine IE compared to 3.5% (*n* = 6/171) of veterinarians in corporate practice. This difference was statistically significant (*p* = 0.05).

### Canine Idiopathic Epilepsy Diagnosis and Management

Participants from independent practices scored lower on the question regarding criteria used to diagnose canine IE (mean = 4.0 out of 7) when compared to corporate practice (mean = 4.5 out of 7). This difference was statistically significant (*p* = 0.028).

The mean score for the question regarding when a participant initiates use of ASDs in canine IE was 2.1 out of 4. No statistically significant differences were found between when a participant initiates use of ASDs and the demographic categories.

87.8% of participants (*n* = 206/235) prescribe phenobarbital as their first line long term maintenance ASD. Eleven percent (*n* = 26/235) prescribe imepitoin, 0.8% (*n* = 2/235) prescribed levetiracetam and 0.4% (*n* = 1/235) prescribe diazepam. No statistically significant differences were found between first line long term maintenance ASD and the demographic categories.

**Figure 2:**
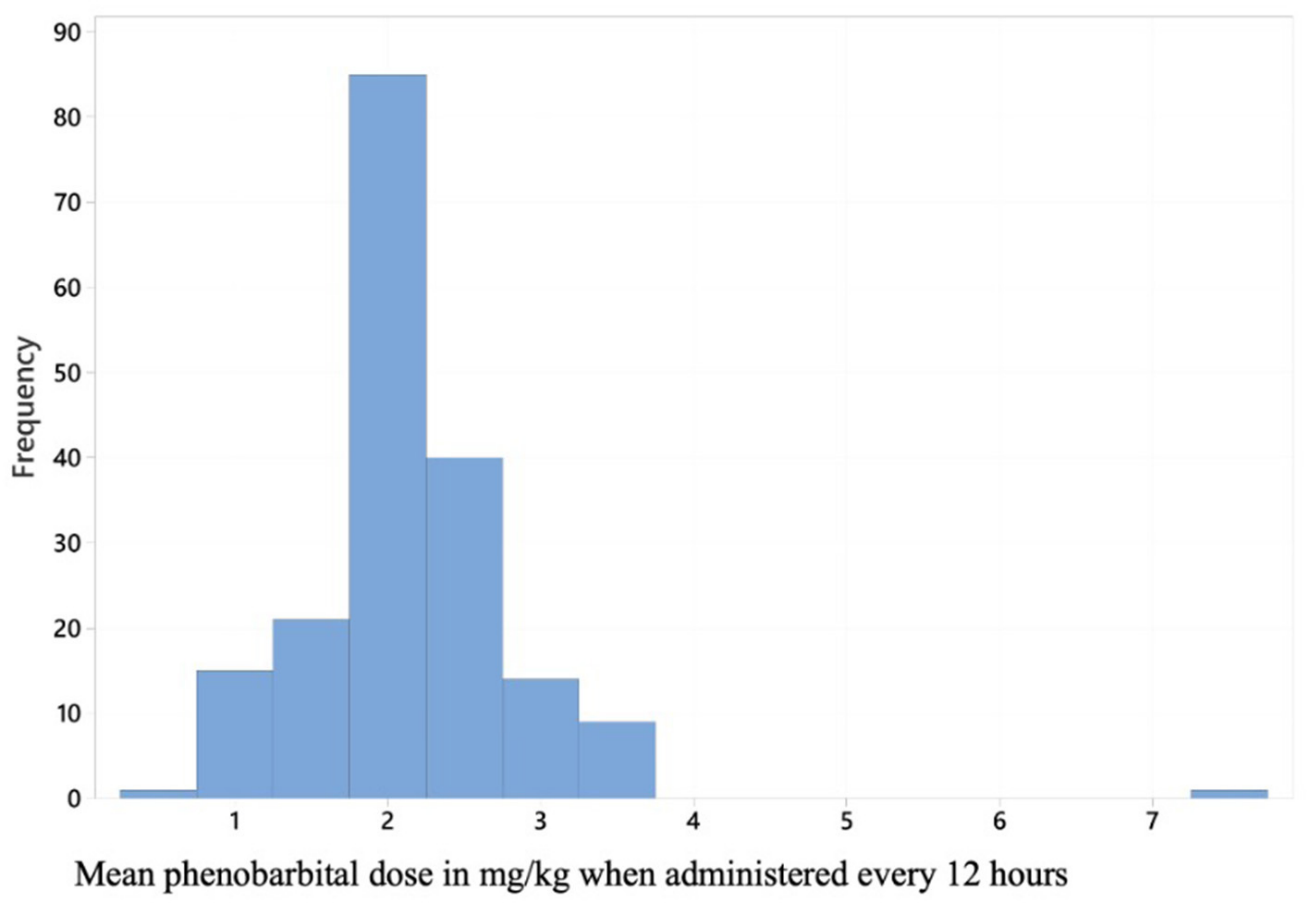
A histogram displaying initial phenobarbital dose reported by the 206 respondents who selected phenobarbital as their preferred first line antiseizure drug for canine idiopathic epilepsy.

As the majority (88%) of participants selected phenobarbital as the preferred first line maintenance ASD, further data analysis was conducted in this subgroup. Of the 206 veterinarians who selected phenobarbital as their preferred first line ASD, 90.2% (*n* = 186/206) provided the initial dosage (dose in mg/kg and administration frequency in hours) that they commonly prescribed in long term maintenance when initiating ASDs. This was an open question where respondents could write the dose in mg/kg and administration frequency in h. The reported mean phenobarbital dosage was 2.1 mg/kg (standard deviation = 0.71) every 12 h (Figure 2). An initial phenobarbital dosage between 2.5 and 3 mg/kg every 12 h (which is recommended by the IVETF) (13) was used by 28.5% (*n* = 53/186) of participants who selected phenobarbital and provided a dose. No statistically significant differences were found between reported phenobarbital dose and the demographic categories.

When questioned about the use of intermittent pulsed treatment with levetiracetam alongside the preferred first-line long-term maintenance ASD, 33.2% (*n* = 78/235) of participants did not use intermittent pulsed treatment with levetiracetam, 26.0% (*n* = 61/235) often used this, and 39.6% (*n* = 93/235) occasionally used this. 1.2% (*n* = 3/235) of participants declined to answer this question. No statistically significant differences were found between demographic categories and the use of intermittent pulsed treatment with levetiracetam.

Forty-six percent of participants (*n* = 107/235) prescribed potassium bromide as a second line long term ASD, 32.4% (*n* = 76/235) prescribed levetiracetam and 11.0% (*n* = 26/235) prescribed imepitoin. 10.6% (*n* = 25/235) of participants who did not use phenobarbital as their first line long term ASD used phenobarbital as their second line long term ASD. There was a difference when comparing second line ASD choice with years working as a veterinarian in clinical practice, with those with more years in practice being statistically significantly more likely to select potassium bromide (*p* = 0.001).

The mean score for the question regarding which criteria veterinarians use to establish if treatment with ASDs has been successful in dogs with IE was 2.98 out of 6. No statistically significant differences were found between which criteria are used to establish if treatment with ASDs has been successful and the demographic categories.

Fifty-three percent of participants (*n* = 125/235) did not recommend any additional treatments for canine IE. Of the remaining 47.0% (*n* = 110/235) recommendations included Purina Neurocare diet (23.4%, *n* = 55/235), environmental modification (12.8%, *n* = 30/235), medium chain triglyceride supplement (6.8%, *n* = 16/235), omega 3 fatty acids (6.4%, *n* = 15/235), other nutraceuticals (3.4%, *n* = 8/235), cannabinoids (2.9%, *n* = 7/235), acupuncture (1.7%, *n* = 4/235), homeopathy (0.8%, *n* = 2/235), physical therapy (0.8%, *n* = 2/235), and skullcap and valerian (0.8%, *n* = 2/235). Participants from small animal practice were statistically significantly more likely to recommend medium chain triglyceride supplements (*p* = 0.034) and those from mixed practice were statistically significantly more likely to recommend Purina Neurocare diet (*p* = 0.037). Environmental modification was statistically significantly more likely to be recommended by participants who had fewer years working in clinical practice (*p* = 0.009).

Neutering in canine IE was recommended by 53.2% (*n* = 125/235) of participants and was not recommended by 46.8% (*n* = 110/235). No statistically significant differences were found between neutering recommendation and demographic categories.

### Perceived Challenges and Educational Needs

**Figure 3:**
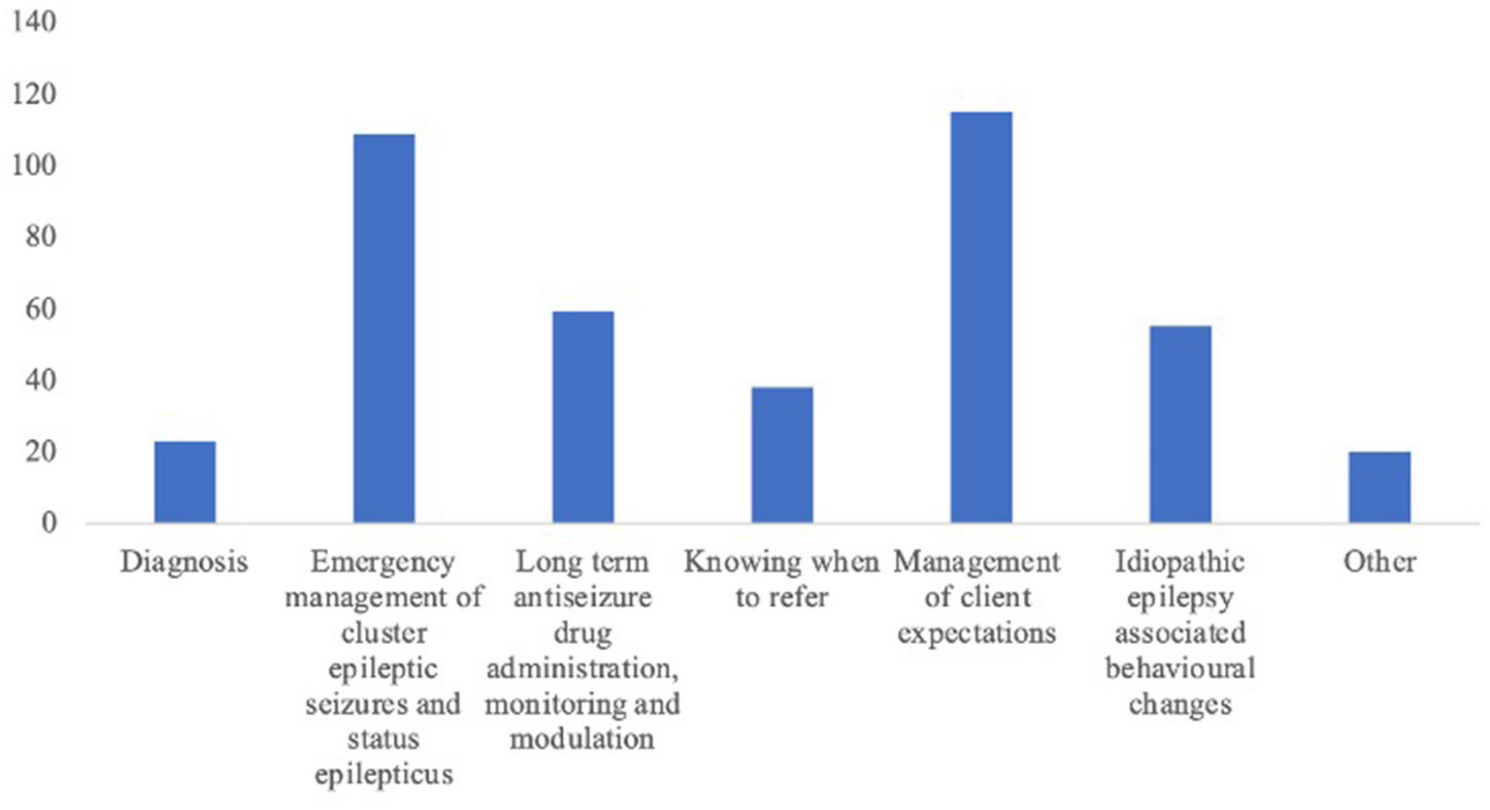
A bar chart displaying which aspects of canine idiopathic epilepsy the 235 respondents felt most challenging.

Forty-nine percent of participants selected managing client expectations (*n* = 115/235), and 45.1% (*n* = 109/235) selected emergency treatment of cluster epileptic seizures and status epilepticus (Figure 3) as the most challenging aspects of canine IE management. Participants from small animal practice with 24-h care were statistically significantly less likely to select emergency treatment of cluster epileptic seizures and status epilepticus as the most challenging aspect of canine IE management (*p* = 0.039). Participants with fewer years' experience were statistically significantly more likely to select long term routine ASD treatment monitoring and modulation as the most challenging aspect of canine IE management (*p* = 0.003).

Respondents sought information about canine IE from multiple sources, including neurology specialists (73.6%, *n* = 173/235), other primary care veterinarians (51.9%, *n* = 122/235), peer reviewed literature (51.9%, *n* = 122/235), neurology books (35.7%, *n* = 84/235), university education (35.7%, *n* = 84/235), advice from technical advisors of the relevant pharmaceutical company (25.1%, *n* = 59/235), non-peer-reviewed veterinary literature (28.5%, *n* = 67/235), internet search engines (14.0%, *n* = 33/235), practice policy (12.3%, *n* = 29/235) and social media (2.1%, *n* = 5/235). Participants from corporate practice were statistically significantly more likely to seek information from neurology specialists (*p* = 0.02) and peer reviewed veterinary literature (*p* = 0.03). Participants from small animal practices with 24-h care were least likely to seek information from social media and this was statistically significant (*p* = 0.03). Participants with fewer years' experience were statistically significantly less likely to seek information from other primary care veterinarians (*p* < 0.001), social media (*p* < 0.001) or use university education (*p* = 0.002). Participants with more years' experience were statistically significantly more likely to seek information from technical advisors of pharmaceutical companies.

**Figure 4:**
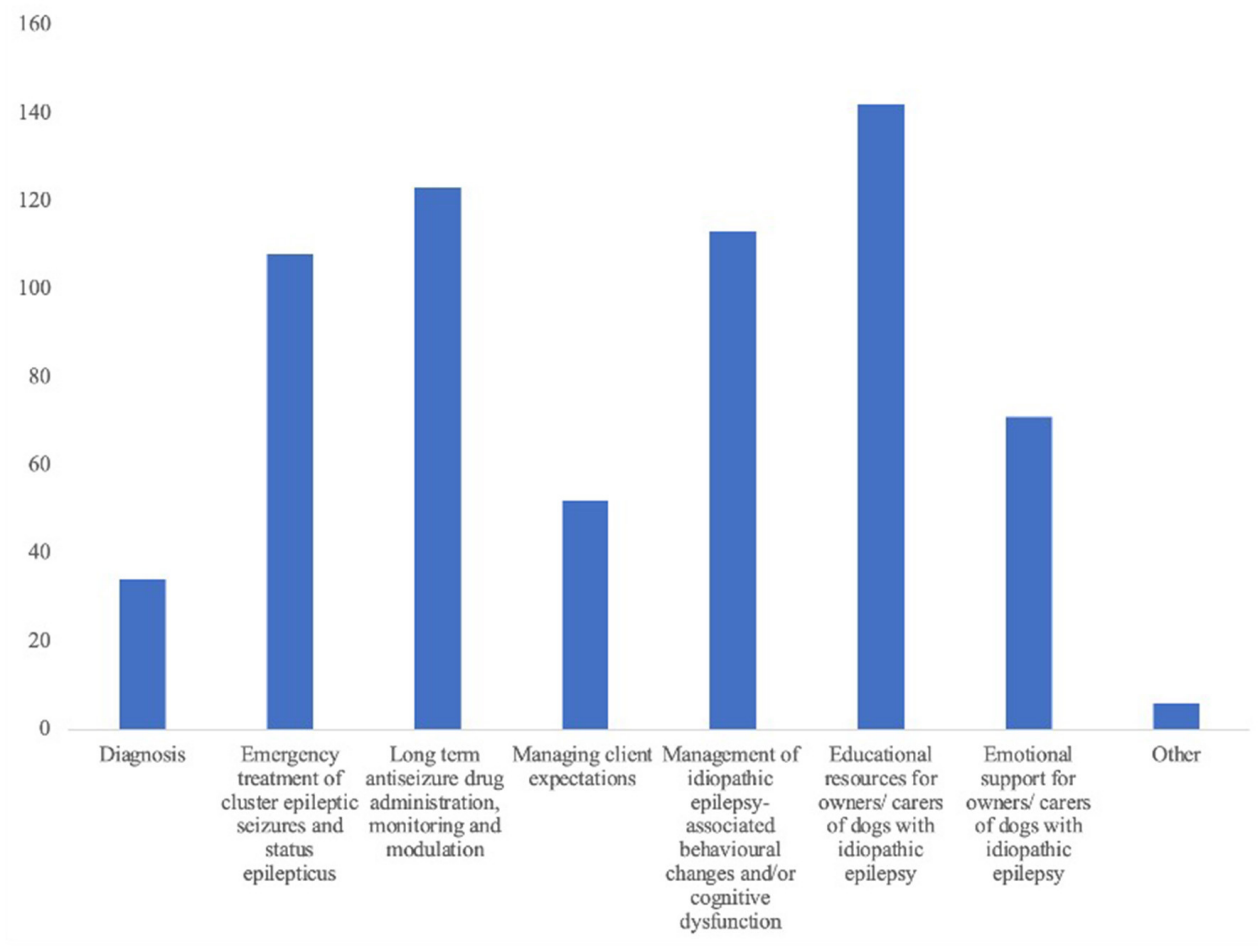
A bar chart displaying in which aspects of canine idiopathic epilepsy the 235 respondents would like more training and support to be provided.

Sixty percent of participants (*n* = 142/235) selected educational resources for owners/carers of dogs with IE as the area where they would like more educational support, 52.3% (*n* = 123/235) selected long term ASD treatment, 48.1% (*n* = 113/235) would like more support in the management of neurobehavioral co-morbidities of canine IE, and 46.0% (*n* = 108/235) would like more support in the management of epilepsy emergency circumstances (Figure 4). Participants with fewer years' experience were statistically significantly more likely to want more educational support in the diagnosis of canine IE (*p* = 0.018) and long-term routine ASD treatment monitoring and modulation (*p* = 0.047).

**Figure 5:**
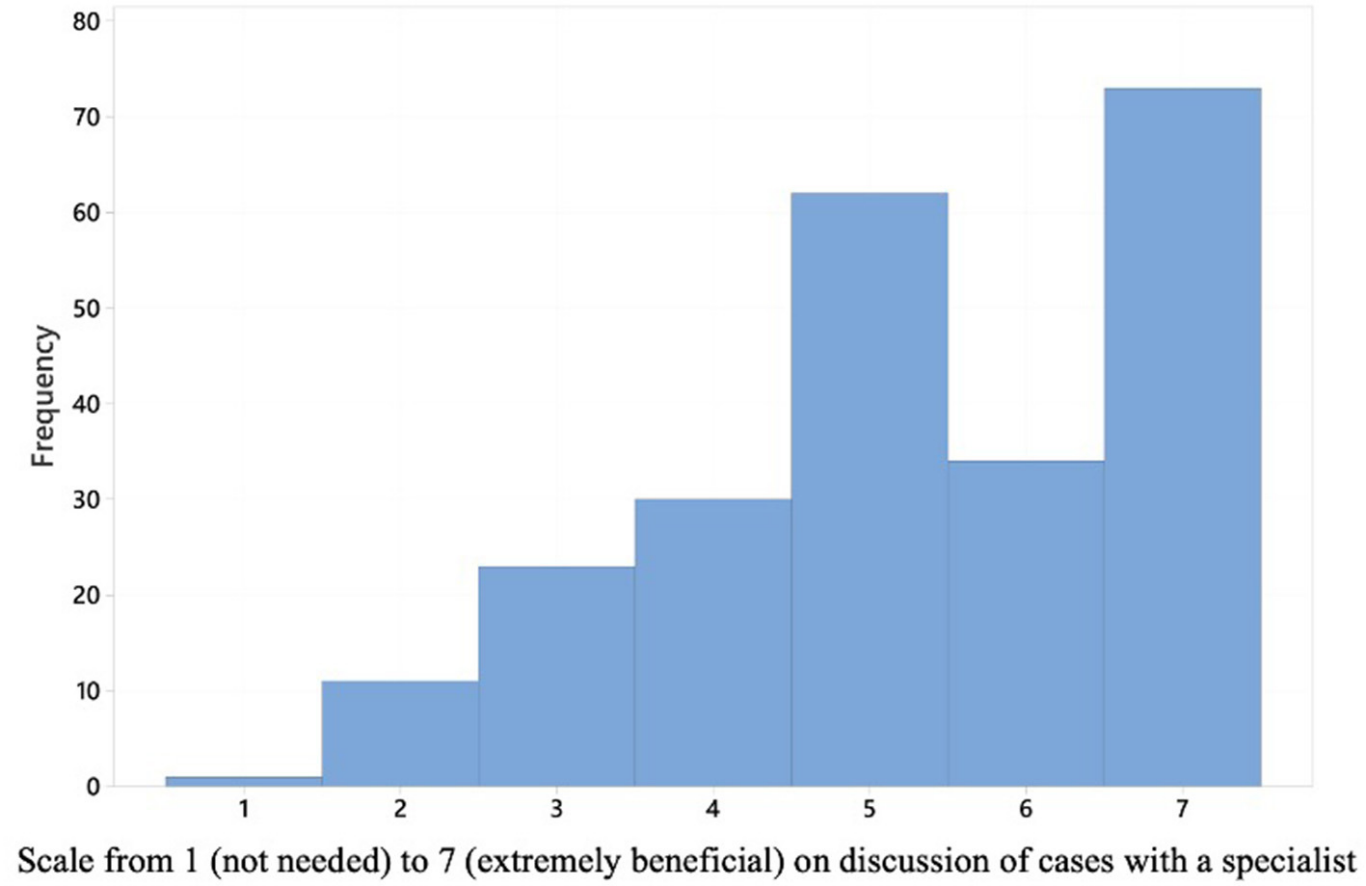
A histogram displaying how the 235 respondents scored on a scale from 1 (not needed) to 7 (extremely beneficial) how beneficial would it be for them to be able to discuss canine idiopathic epilepsy cases with a specialist in veterinary neurology.

The mean score on a scale from 1 (not needed) to 7 (extremely beneficial) for how beneficial it would be for participants to be able to discuss canine epilepsy cases with a specialist in veterinary neurology was 5.3 (standard deviation = 1.5) (Figure 5). This was statistically significantly negatively correlated (*p* = 0.014) with number of years in clinical practice. The more years in practice a respondent had spent, the less beneficial they scored discussion of canine IE cases with a specialist in veterinary neurology.

## Discussion

This is the first study investigating how primary care veterinarians in the UK manage canine IE and which resources and support they feel would be beneficial to them.

Phenobarbital is a widely used ASD in veterinary medicine due its efficacy, low cost, safety, and variety of formulations (13, 15, 16). In our study, 87.8% (*n* = 206/235) of participants reported that phenobarbital was the first line ASD most frequently used for long term treatment of canine IE. A cross-sectional survey investigating ASD preferences amongst Australasian veterinarians in 2009 (before imepitoin was introduced to the Australasian market)^4^, reported that phenobarbital was used by 99% (*n* = 177/178) of participants for managing canine epilepsy and by 94% (*n* = 119/127) of participants for feline epilepsy (18). In a cross-sectional survey on ASD use in dogs with suspected IE among board-certified emergency (*n* = 128) and neurology specialists in North America (where imepitoin is not licensed), phenobarbital was the initial ASD of choice for 66% (*n* = 114/172) of neurologists and 64% (*n* = 82/128) of emergency and critical care specialists (19). In another web-based, cross-sectional survey (20) of Dutch primary care veterinarians working in small animal or mixed-practice in 2016, phenobarbital was used by 89% (*n* = 91/102) and imepitoin was used by 75% (*n* = 77/102) respondents in the management of cases of canine epilepsy. This survey (20) did not ask about preferred first line ASD for long term treatment of canine IE.

The reported efficacy of phenobarbital monotherapy in reducing or leading to remission of epileptic seizure occurrence in dogs with serum phenobarbital concentration within the recommended target range (20–30 mg/L) varies between 60 and 93% (13, 21–25). The mean dose of phenobarbital used by respondents was 2.1 mg/kg every 12 h which is consistent with recommendations by phenobarbital manufacturers and the BSAVA Small Animal Formulary (1–2.5 mg/kg/every 12 h) (26, 27). However, the IVETF recommend a starting dose of 2.5–3 mg/kg every 12 h based on phenobarbital pharmacokinetic studies and serum phenobarbital concentrations required to achieve seizure control in most dogs (13). Only 22.6% (*n* = 53/235) participants in our study reported 2.5–3 mg/kg every 12 h as the most used initial phenobarbital dose. This suggests the common point of reference are the product data sheets rather than IVETF consensus guidelines on treatment. More education on initial phenobarbital dosage and target serum phenobarbital concentrations may be beneficial in primary care.

IVETF guidelines were published in 2015 to provide guidance on how to diagnose canine IE, when to start treatment with ASDs, which ASDs are safe and efficacious, recommended ASDs' dosage and monitoring as well as definitions of treatment success (12–14, 28). These guidelines are based on evidence-based literature, the cascade, and the authors experiences. When considering how closely participants aligned with IVETF guidelines on the topics of diagnosis, ASD initiation and outcome assessment, on average participants would score around half of the available points. This could suggest that primary care veterinarians may have only moderate awareness of IVETF guidelines or that they find it challenging to implement them in their work setting. The guidelines themselves form detailed, lengthy documents which may seem inaccessible to read and use in a busy primary care practice. Respondents from independent veterinary practices scored significantly lower than respondents from corporate veterinary practices about diagnosis of canine IE. This may be due to structured continued professional development programs or facilitated access to veterinary neurologist specialists working in referral centers. There was no difference in ability to diagnose and treat cases based on the number of years working in clinical practice, whether the participants had access to 24-h care and whether they worked in small animal or mixed practice.

The topic of neutering in the management of canine IE remains controversial (29) and this may be the reason why 53.2% (*n* = 125/235) of participants recommended neutering and 46.8% (*n* = 110/235) did not. No statistically significant associations were found between neutering recommendation and the different demographic categories. Two studies suggest that survival is longer in dogs with IE that are not neutered (30, 31). A VetCompass study in the UK investigated associations of neutering with IE in 117 Labrador retrievers and 57 Border collies diagnosed with IE in primary care. The majority (74.2%) of neutered dogs were neutered prior to the onset of IE. Dogs intact at IE onset had longer median survival times than dogs neutered before IE onset (males, 1,436 days vs. 1,234 days: females, 1,778.5 days vs. 1,261 days) (30). In a retrospective study in Denmark, survival was significantly shorter in neutered male dogs with IE (median survival: 38.5 months) compared to intact male dogs with IE (median survival: 71 months) (31). Cluster seizures in dogs with IE have been associated with a reduced likelihood of achieving seizure freedom, decreased survival time and increased likelihood of euthanasia. A retrospective study including 384 dogs with IE treated at a multi-breed canine specific epilepsy clinic, did not identify any association between occurrence of cluster epileptic seizures and sex or neuter status (32). In contrast, in a previous retrospective study (33) including 407 dogs with IE, intact males were twice as likely than neutered males to suffer from cluster seizures and intact females had significantly more frequent cluster seizures than neutered females. The lack of a clear indication from the evidence-based literature either in favor or opposing neutering in dogs with IE may be one of the reasons why the population of primary care veterinarians in this study is split down the middle when it comes to this difficult to navigate topic.

53.2% (*n* = 125/235) of participants did not recommend any additional treatments for canine IE beyond use of ASDs. When additional treatments were recommended environmental modification, Purina Neurocare diet and medium chain triglyceride supplements were the most frequently selected. Participants with fewer years' experience working as a veterinarian were more likely to recommend environmental modification. Dogs with IE can present with concurrent behavioral and cognitive abnormalities, which in some cases can become severe following the onset of IE (7–11). Dogs can become more anxious and fearful in nature (7). The cognitive and behavioral abnormalities can be present every day and may have a significant impact on the dog's and caregiver's quality of life. While changing the environment will not directly benefit seizure control (unless stress is a trigger for the seizures) it can be used to address the IE-associated co-morbidities and significantly enhance the care delivered to dogs with IE (9, 34). An online questionnaire study investigated how owners of dogs with IE manage their dog's interictal anxiety (34). This cross-sectional study showed that while 83.6% of owners reported to be using a range of approaches to manage interictal anxiety there is a lack of consistent information on how to do this and lack of support from veterinary professionals (34). There is scope that by providing better education to veterinarians on this topic that better support could be provided to the owners of dogs with IE which could lead to significant advancements in the care of these patients.

Participants from small animal primary care were more likely to recommend medium chain triglyceride supplements than those from mixed primary care practice. There is some evidence to support the use of medium chain triglycerides as a dietary supplement in drug resistant cases of canine IE (35–38). There was a small decrease in seizure frequency of some dogs in a recent randomized controlled trial (37). Currently the most effective dose of medium chain triglycerides and their ideal formulation for use in canine IE is unknown.

While dietary manipulation is commonplace by owners of dogs with IE (two thirds of owners changed the diet following IE diagnosis in one study (37) it is important that dogs with IE are fed a high-quality diet due to the long-term nature of management (37–39). A low-calorie diet is often necessary to maintain a healthy body weight in dogs with IE and long-term ASD treatment (38). Medium chain triglyceride oils may be high in fat and powders may be high in carbohydrates. While cheap and widely available products which contain medium chain triglyceride may vary greatly in the quality and quantity found within. Factors to bear in mind include that restriction of protein or fat can increase clearance and elimination of phenobarbital (39). While most dietary supplements are considered safe products, administering large quantities of a medium chain triglyceride oil may lead to gastrointestinal adverse reactions (40, 41). When administering a medium chain triglyceride oil the total diet composition should be carefully considered as there may be risk of pancreatitis associated with hypertriglyceridemia (42). Therefore, this should be done in consultation with a veterinary nutrition specialist to feed a complete and balanced diet with adequate quantities of proteins, fats, vitamins, and minerals. Participants from mixed primary care were more likely to recommend the Purina Neurocare diet. While the Purina Neurocare diet does not have a label claim for the management of canine IE, it does contain medium chain triglycerides (42).

It is promising that veterinary neurology specialists are the most common source of information about management of canine IE. Participants from corporate practices were more likely to seek information from veterinary neurology specialists and this may arise from greater access to veterinary neurology specialists if, for example, the corporate practice owns both primary care and referral care practices. This may provide an opportunity to pass on invaluable information about evidence-based case management, including the IVETF consensus guidelines and recent scientific publications. Concerningly, as the mean years' experience in practice increased participants were more likely to seek information from pharmaceutical companies which may differ from advice provided by veterinary neurology specialists.

In this study, participants found managing client expectations to be most challenging alongside emergency management of cluster seizures and status epilepticus. Participants from small animal practice with 24-h care were statistically significantly less likely to select emergency management as the most challenging aspect of canine IE management. Participants with fewer years' experience in practice were more likely to select long term routine ASD treatment monitoring and modulation as the most challenging aspect of canine IE management. This may reflect the lack of experience gained from case continuity and following a case of canine IE over several years and learning from the management of these cases. These participants also wanted more educational support in the diagnosis of canine IE and long-term routine ASD treatment monitoring and modulation. The participants overall indicated they would like more educational support in the management of cluster seizures and status epilepticus, as well as in the long-term treatment of canine IE and in the management of canine IE related co-morbidities. It is the authors' opinion that the provision of better educational resources and of support in the long-term management of canine IE will allow better management of client expectations.

The main limitation of this study is the relatively low response rate and therefore the results may not reflect the entire small animal veterinary profession in the UK. There are an estimated 27,200 veterinarians employed in the UK (43), of which, 53% work exclusively in small animal practice and 12% work in mixed practice (44). The invite to participate in the study was shared in the electronic newsletter received by 655 primary care veterinary surgeons within Linnaeus as well as widely and repeatedly promoted by various UK veterinary media during nearly 4 months. The relatively low response rate may be due challenges in practice to find free time to spend 5–10 min to undertake a survey. Primary care veterinarians in the UK are under immense pressure on a day-to-day basis with the number of pet dogs in the UK increasing from 7.6 million in 2010–2011 to 12.5 million in 2020–2021 (45). This increase applies significant time pressures in primary care practices. During the study period, the veterinary profession in the UK has also been under exceptional stress (46) due to staff shortages from COVID-19 combined with the increased workload (47). However, despite this, the results of this study provide insight into how cases of canine IE are managed in primary care and represent a starting point to inform educational resources and support strategies to improve quality care of canine IE in primary care.

## Data Availability Statement

The raw data supporting the conclusions of this article will be made available by the authors, without undue reservation.

## Ethics Statement

The studies involving human participants were reviewed and approved by Ethics Review Panel of the Royal College of Veterinary Surgeons (2021-75-GRIFFIN, approval granted 1st of October 2021). The patients/participants provided their written informed consent to participate in this study.

## Author Contributions

LD, SG, and FS contributed to conception and design of the study. LD and SG conducted data analysis with the support of a statistician. SG set up the online survey, revised and coded responses, and wrote the first draft of the manuscript with LD's support. All authors contributed to manuscript revision, read, and approved the submitted version.

## Funding

Linnaeus Veterinary Limited supported the costs of the Open Access Publication Charges.

## Conflict of Interest

SG, FS, and LD were employed by Linnaeus Veterinary Limited. FS and LD have received consultancy fees and/or speakers fee from pharmaceutical companies producing antiseizure drugs. These activities are unrelated to the conduct of this study.

## Publisher's Note

All claims expressed in this article are solely those of the authors and do not necessarily represent those of their affiliated organizations, or those of the publisher, the editors and the reviewers. Any product that may be evaluated in this article, or claim that may be made by its manufacturer, is not guaranteed or endorsed by the publisher.

## Footnotes

^1^Vmd.defra.gov.uk. 2021. Available at: https://www.vmd.defra.gov.uk/productinformationdatabase/files/SPC_Documents/SPC_141082.PDF (accessed 30 September 2021).

^2^Noahcompendium.co.uk. 2021. *Libromide® 325 mg Tablets for Dogs*. Available at: https://www.noahcompendium.co.uk/?id=-449908&template=template_printview (accessed 30 September 2021).

^3^European Medicines Agency. 2021. *Pexion - European Medicines Agency*. Available at: https://www.ema.europa.eu/en/medicines/veterinary/EPAR/pexion (accessed 30 September 2021).

^4^ https://www.tga.gov.au/sites/default/files/scheduling-decisions-1407-final.pdf
